# Pharmacodynamic studies of taniborbactam (VNRX-5133) combined with cefepime against β-lactamase–producing Gram-negative bacteria in a neutropenic murine thigh infection model

**DOI:** 10.1093/jac/dkaf431

**Published:** 2025-12-05

**Authors:** Panagiota-Christina Georgiou, Maria Siopi, Marilena Tsala, Claudia Lagarde, Wendy Kloezen, Anouk E Muller, Joseph Meletiadis

**Affiliations:** Clinical Microbiology Laboratory, Attikon University General Hospital, Medical School, National and Kapodistrian University of Athens, Rimini 1, Haidari, Athens 12462, Greece; Clinical Microbiology Laboratory, Attikon University General Hospital, Medical School, National and Kapodistrian University of Athens, Rimini 1, Haidari, Athens 12462, Greece; Clinical Microbiology Laboratory, Attikon University General Hospital, Medical School, National and Kapodistrian University of Athens, Rimini 1, Haidari, Athens 12462, Greece; Department of Medical Microbiology Radboud University, Nijmegen Medical Center, Nijmegen, The Netherlands; Department of Medical Microbiology and Infectious Diseases, Erasmus Medical Center, Rotterdam, The Netherlands; Department of Medical Microbiology and Infectious Diseases, Erasmus Medical Center, Rotterdam, The Netherlands; Department of Medical Microbiology, Haaglanden Medisch Centrum, The Hague, The Netherlands; Clinical Microbiology Laboratory, Attikon University General Hospital, Medical School, National and Kapodistrian University of Athens, Rimini 1, Haidari, Athens 12462, Greece; Department of Medical Microbiology and Infectious Diseases, Erasmus Medical Center, Rotterdam, The Netherlands

## Abstract

**Background:**

Taniborbactam (VNRX-5133) is a novel boronate-based β-lactamase inhibitor that directly inhibits all four classes of β-lactamases. We studied the pharmacodynamics of taniborbactam in combination with cefepime against β-lactamase–producing Enterobacterales and *Pseudomonas aeruginosa*.

**Methods:**

*In vitro*, cefepime/taniborbactam combination was assessed with a checkerboard broth microdilution method against two ESBL-producing Enterobacterales and one AmpC- and one VIM-producing *P. aeruginosa* isolates (cefepime MIC 16–256 mg/L). *In vivo*, neutropenic infected mice were treated with cefepime, every 2 h for 24 h, alone or in combination with taniborbactam at q2h, q4h and q8h dosing intervals. Single dose escalation and dose-fractionation experiments were conducted in order to describe plasma pharmacokinetics and pharmacodynamics of taniborbactam, respectively.

**Results:**

*In vitro*, reversal of phenotypic resistance to cefepime was found at taniborbactam ≤0.03 and 0.25 mg/L for ESBL-producing *E. coli* and *K. pneumoniae*, and at 0.125 and 2 mg/L for VIM- and AmpC constitutively-producing *P. aeruginosa*, respectively. *In vivo*, cefepime alone marginally reached stasis against Enterobacterales and AmpC-producing *P. aeruginosa*. Taniborbactam restored cefepime’s static effect against all isolates and its 1 log_10_ kill effect against all strains except the *K. pneumoniae* isolate. The percentage of time above free concentration threshold (%*f*T > Ct) best described taniborbactam efficacy (*R*^2^ = 0.50–0.80). At high cefepime exposures, area under the free concentration–time curve (*f*AUC) performed equally well (*R*^2^ 0.49–0.70). A 40%–50% and 60%–100% *f*T > Ct of taniborbactam was associated with stasis and 1 log_10_ kill, respectively, at taniborbactam concentrations where reversal of cefepime resistance was found *in vitro*.

**Conclusions:**

Taniborbactam restored cefepime’s activity against resistant Gram-negative bacteria in a time- and concentration-dependent manner at low and higher cefepime exposures, respectively.

## Introduction

Bacterial infections are threatening modern public health despite the advances of science.^1,2^ Bloodstream bacterial infections are among the top seven causes of death in North America and Europe.^2^ Increasing rates of antibiotic resistance, and particularly of MDR bacteria, limit therapeutic options and increase mortality by those infections.^3^ As β-lactam drugs play a major role in treating those infections, there is great interest in overcoming resistance to these drugs, which is usually associated with the production of β-lactamases, enzymes that degrade β-lactam drugs. Among the WHO’s list of bacteria for which new antibiotics are urgently needed are ESBL-producing and carbapenem-resistant *Pseudomonas aeruginosa* and Enterobacterales.^4^ In order to overcome β-lactam resistance, β-lactamase inhibitors have been used successfully combined with β-lactam drugs for more than 70 years.^5^ Many combinations of β-lactam drugs and inhibitors of β-lactamases have been developed to overcome resistance to β-lactam drugs, thereby increasing their activity, such as piperacillin/tazobactam,^6^ cefepime/tazobactam^7^ and ceftazidime/avibactam.^8,9^

Cefepime is a fourth-generation cephalosporin that rapidly crosses the outer membrane of Gram-negative bacteria via porins,^10,11^ has broad-spectrum activity against both Gram-negative and Gram-positive bacteria,^10,12^ and has been combined with tazobactam in order to increase its activity against β-lactamase–producing isolates.^7,13^ However, tazobactam is already more than 30 years old and has lost its broad-spectrum effectiveness. Resistance to the combination of cefepime/tazobactam has emerged.^14^ Taniborbactam (formerly VNRX-5133) is a new cyclic boronate β-lactamase inhibitor, in development by VenatoRx Pharmaceuticals Inc.,^15,16^ and has broad-spectrum activity against Ambler class A ESBLs, class A enzymes (*Klebsiella pneumoniae* carbapenemase), class B (Verona integron-encoded metallo-β-lactamase [VIM] and New Delhi metallo-β-lactamase),^17^ class C (AmpC) and class D enzymes.^18,19^ Inhibition of serine β-lactamases by taniborbactam occurs by the formation of a reversible covalent bond with the enzyme whereas MBLs are inhibited competitively. *In vitro* studies showed that the combination of cefepime/taniborbactam was most effective even compared with ceftazidime/avibactam.^20,21^ The antibacterial activity of cefepime was restored resulting in a marked decrease of MICs of resistant strains.^20,22^

We therefore investigated the exposure–response relationship of cefepime alone and in combination with taniborbactam against two Enterobacterales (*Escherichia coli* and *K. pneumoniae*) and two *P. aeruginosa* isolates harbouring different β-lactamases (ESBL, AmpC and MBL) in a neutropenic murine thigh infection model. The pharmacokinetic/pharmacodynamic (PK/PD) index that best described cefepime/taniborbactam activity was also determined.

## Methods

### Bacterial isolates

Four previously well-characterized cefepime-resistant isolates, namely one ESBL-producing *E. coli*, one ESBL-producing *K. pneumoniae* and two *P. aeruginosa* (one VIM- and one AmpC constitutively -producing), were used. The MICs of cefepime alone and in combination with taniborbactam were determined in an *in vitro* checkerboard study by the broth microdilution method.^23^

### Drugs

Taniborbactam (VenatoRx Pharmaceuticals Inc.) was solubilized in PBS pH  5, at 50 mg/mL or 100 mg/mL, according to the manufacturer’s instructions. Cefepime (Bristol-Myers Squibb Company) was dissolved in saline at a stock concentration of 100 mg/mL. The solutions were stored at −80°C until used and were combined with and/or diluted in normal saline to the final concentrations needed for the experiments.

### Infection model

All animal procedures were carried out in the Animal Facility for Medical and Scientific Purposes of the University General Hospital Attikon (EL 25 BIO 014), Athens, Greece. The animal studies were conducted in accordance with the recommendations of the European Community [Directive 86/609/EEC, 24 November 1986 and 2010/63/EE, 2010 (276/33/20/10.2010)]. The studies were approved by its Animal Welfare Committee (no. 6542/17-12-2015). Outbred female CD-1 mice, obtained by the Hellenic Pasteur Institute, Greece, and Charles River Laboratories Models and Services, Germany, were used at the age of 7–8 weeks, weighing 20–25 g. The animals were allowed to acclimatize for at least 5 days upon arrival and were housed under standard conditions with water and food supplied *ad libitum*. Neutropenia was induced by two intraperitoneal injections of cyclophosphamide, at Day −4 (150 mg/kg) and at Day −1 (100 mg/kg) prior to infection, and mice were examined for welfare once daily after immunosuppression. Infection was induced (Day 0) by intramuscular injection of 0.05 mL bacterial inoculum of approximately 10^5^–10^6^ cfu/thigh.

### Pharmacokinetic studies

Single doses of cefepime and taniborbactam were injected subcutaneously (s.c.) in infected mice, for six 2-fold increasing cefepime and taniborbactam doses 4–128 mg/kg and 2–64 mg/kg, respectively. The dose was administered s.c. (0.1 mL) 2 h after infection (t = 0 h). Blood samples were collected in 1 mL K3EDTA tubes through orbital sinus bleeding under isoflurane anaesthesia. Animals were immediately euthanized through cervical dislocation and blood samples taken at timepoints 0, 0.08, 0.25, 0.5, 0.75, 1, 1.5, 2, 3, 4, 6, and 8 h after single dose injection. Blood samples were centrifuged immediately after collection in a pre-cooled centrifuge, and plasma was stored at −80°C. For each timepoint two animals were euthanized in order to have duplicates for each condition. The concentrations of cefepime and taniborbactam in plasma were determined by LC-MS/MS by Venatorx. The limit of detection for the total concentrations of both cefepime and taniborbactam was 1 ng/mL. Protein binding for cefepime and taniborbactam was determined in the equilibrium dialysis chamber and analysed via LC-MS/MS by Venatorx.

### Pharmacodynamic studies

Treatment was administered subcutaneously using 0.1 mL solutions containing the appropriate concentration of cefepime alone and in combination with taniborbactam or saline (placebo) starting at t = 0 h (2 h after infection) for 24 h. Two-fold increasing cefepime total daily dose (TDD) doses 48–1536 mg/kg administered q2h were used in order to determine the dose–response relationship of cefepime alone. A marginally ineffective cefepime q2h dosing regimen was then combined with 2-fold increasing TDD doses of taniborbactam 0.38–768 mg/kg administered at q2h, q4h and q8h in order to determine the PK/PD index that best described taniborbactam activity. The marginally ineffective dose of cefepime that was used in combination therapy was determined by visual inspection of dose–response curves of cefepime monotherapy and corresponded to the dose just before the dose–response curve drops, i.e. close to effective doses of cefepime. Thus, any reduction in bacterial burden towards stasis and bacterial killing observed with combination therapy would be caused by taniborbactam via inhibiting β-lactamase activity allowing the antibacterial effect of cefepime to be re-exerted. All dosing regimens were performed in at least two animals per dosing regimen.

### Bacterial burden quantitation

At t = 0 h, 2 h after infection, two mice were humanely euthanized to determine the initial number of cfu/thigh just before treatment. All other animals were euthanized at t = 24 h unless the welfare of the animals indicated earlier termination, following animal welfare regulations. Thighs were taken and moved to a pre-cooled 10 mL plastic tube (Transport Tube, Omnilabo, NL) containing 2 mL PBS (NaCl 8.00 g/L, Na_2_HPO_4_ * 2H_2_O 1.44 g/L, KH_2_PO_4_ 0.26 g/L, pH 7.2–7.4). Subsequently, thighs were homogenized using the Ultra-Turrax homogenizer (IKA Labortechnik, Germany). For each thigh 10-fold dilution series were prepared and 3 × 10 µL per dilution were plated (Chromagar, bioMérieux, NL). The following day, colonies were counted and the number of cfu/thigh was calculated. The drug effect was then determined as the difference between the log_10_cfu/thigh values at t = 24 h and t = 0 h (mean value of two mice) expressed as Δlog_10_cfu, i.e. positive result for growth, zero for static effect and negative for reduction of inoculum (killing).

### Analysis

Dose and exposure–response (Δlog_10_cfu) data of cefepime alone and taniborbactam with q2h cefepime were analysed with nonlinear regression analysis using the E_max_ model. In order to find the most effective dosing frequency for taniborbactam, q2h, q4h and q8h dose–response curves were compared statistically by comparing the ED_50_ (effective dose corresponding to 50% of E_max_ − E_min_) using the extra sum-of-squares F test of Graphpad Prism. In cefepime monotherapy experiments, the dose and the percentage of dosing interval that the free concentration remained above the MIC (%ƒT > MIC) for a static and 1 log_10_ kill effect was calculated for each strain. In dose-fractionation experiments with different taniborbactam dosing regimens and the marginally ineffective q2h cefepime doses, the taniborbactam dose, maximum free concentration (*f*C_max_), area under the free concentration–time curve (*f*AUC), and percentage of dosing interval that free concentration remained above different threshold concentrations (%*f*T > Ct) for Ct 0.015 to 4 mg/L were associated with Δlog^_10_^cfu for each strain.

The C_max_ values were calculated from raw data and the AUCs were calculated using the trapezoidal rule. The %*f*T > MIC of cefepime and %*f*T > Ct of taniborbactam were estimated simulating exposures of unbound cefepime and taniborbactam using MicLab 2.36 (Medimatics, Maastricht, The Netherlands) and the population pharmacokinetic parameters. Equations used in the program were the classical polyexponential equations Ci(t) = PolyExp(i, *n*, t), where i = number of the compartments, *n* = number of doses and t = time from last dose (http://www.boomer.org/c/p4/c19/c19.pdf). The %*f*T > Ct was then determined using a bisectional rootfinder for intersections between simulated curve and concentration, and calculating the difference between intersection values. Free drug concentrations were used in all calculations. Protein binding was 20% for cefepime and 19.6% for taniborbactam as determined in the equilibrium dialysis chamber and analysed via LC-MS/MS by VenatoRx Pharmaceuticals Inc. The PK/PD index that described better taniborbactam’s enhancement of cefepime’s activity was determined based on the overlapping q2h, q4h and q8h curves and the highest R^2^ of E_max_ model as previously described.^7,24,25^

## Results

### In vitro susceptibility studies

The MICs of cefepime alone and in combination with taniborbactam are presented in Table 1. All strains were resistant to cefepime (MICs >8 mg/L).^23^ The presence of 4 mg/L taniborbactam decreased the MIC of cefepime to 0.06–4 mg/L for all strains. Reversal of phenotypic resistance to cefepime was observed at ≤0.03 mg/L of taniborbactam for *E. coli*; 0.25 mg/L of taniborbactam for *K. pneumoniae*; 0.125 mg/L of taniborbactam for the VIM-producing *P. aeruginosa* isolate; and 2 mg/L for the AmpC constitutively -producing *P. aeruginosa* strain.

**Table 1.** MICs, characterization, β-lactamase genotypes, and characteristics of Enterobacterales and *P. aeruginosa* strains used in the experiments with cefepime and taniborbactam

Strain no.	Resistance summary	Cefepime MIC (mg/L) alone	Cefepime MIC (mg/L) at the following concentrations (mg/L) of taniborbactam:							
			0.03	0.06	0.125	0.25	0.5	1	2	4
*E. coli* 52	TEM-1, CTX-M 14	32	0.5	0.5	0.25	0.125	0.06	0.125	0.06	0.06
*K. pneumoniae* 59	SHV-1, OXA-1, CTX-M 15	256	64	32	8	2	1	1	1	0.5
*P. aeruginosa* 106	AmpC constitutive expression,	32	32	32	32	16	16	16	8	4
*P. aeruginosa* 132	VIM	16	16	16	8	4	2	1	1	1

### Pharmacokinetics

**Figure 1.**
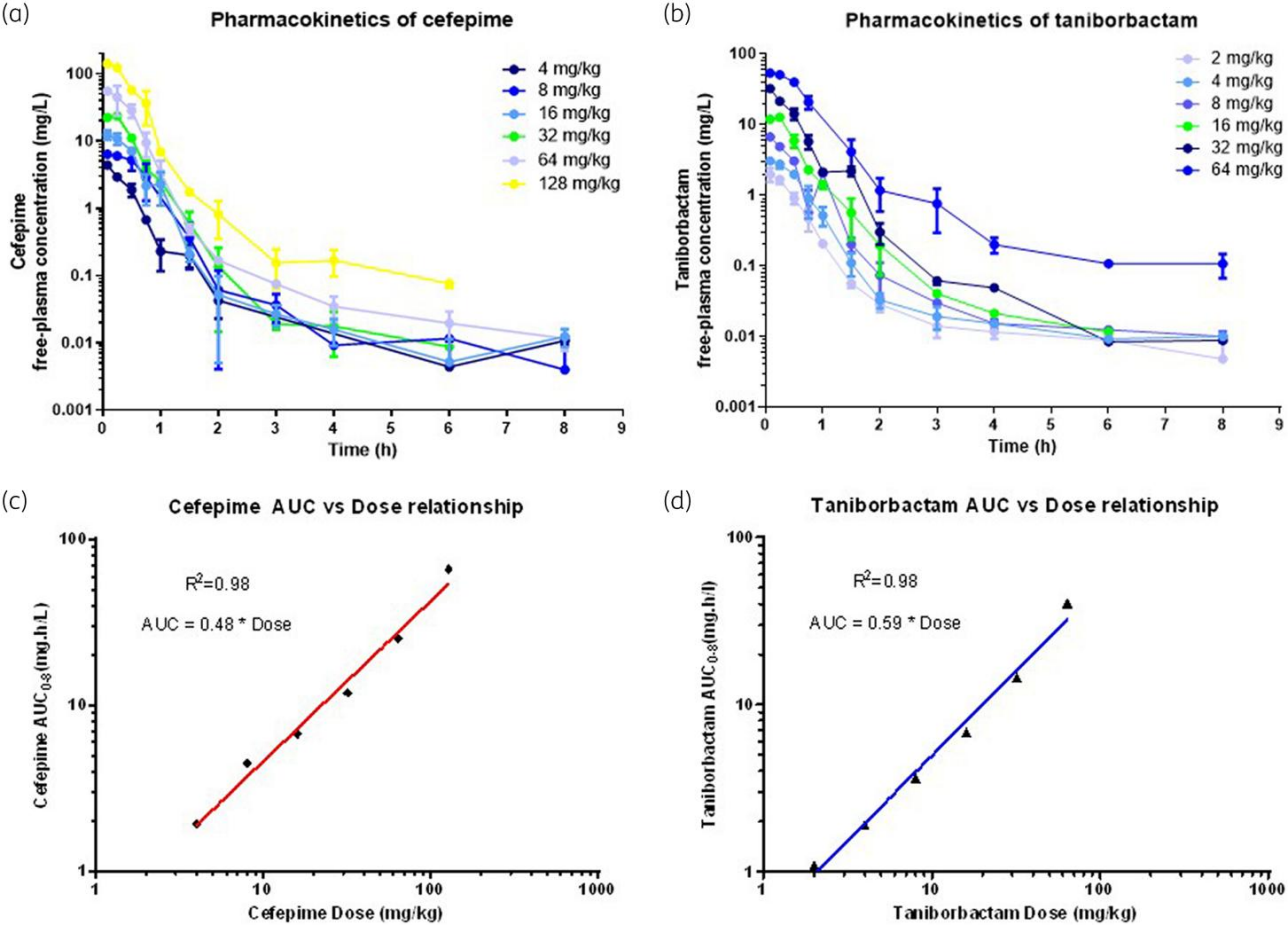
Single dose time–concentration profiles of six 2-fold increasing doses of cefepime (a) and taniborbactam (b) by subcutaneous injection and dose-proportionality of cefepime (c) and taniborbactam (d) in infected mice.

Time–free concentration profiles of cefepime and taniborbactam in plasma are shown in Figure 1a and b. For cefepime, a one-compartment model described well the time–concentration profiles of cefepime in plasma up to 2 h, which is the dosing interval for administering cefepime to mice in pharmacodynamics studies. Serum pharmacokinetics of cefepime followed a one-compartment model with absorbance rate Ka = 15 h^−1^, volume of central compartment Vc = 0.486 L/kg and clearance CL = 3.024 h^−1^, whereas serum pharmacokinetics of Ka is the rate at which a drug enters into the systemVc is the central volume of distribution upon administration Ka = 20 h^−1^, Vc = 0.628 L/kg, volume of peripheral compartment Vp = 0.926 L/kg, CL = 1.49 h^−1^ and intercompartmental clearance Q = 0.109 h^−1^ (Table S1, available as Supplementary data at *JAC* Online). Goodness-of-fit plots did not show significant deviation of the model (Figures S1 and S2). The t_1/2_ of taniborbactam was estimated to be t_1/2a_ = 0.293 h for the central compartment and t_1/2b_ = 0.75 h for the peripheral compartment.

### Pharmacodynamics of cefepime alone

**Figure 2.**
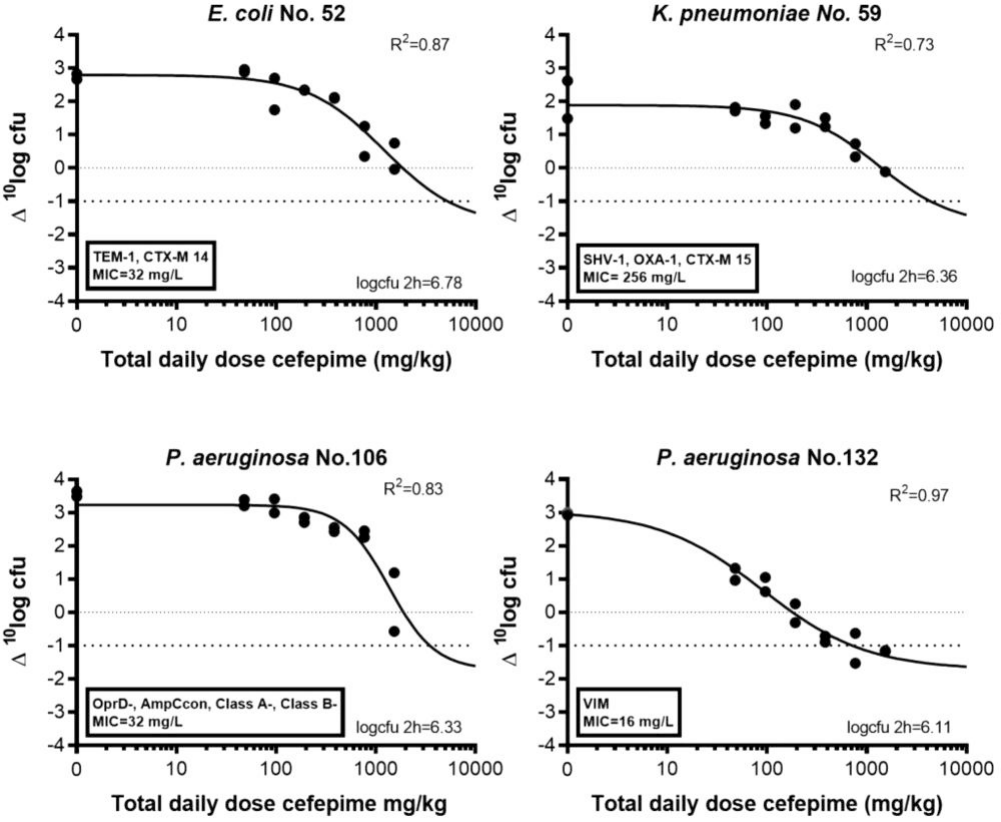
Dose–response relationships of cefepime q2h dosing regimens versus 24 h change in the log_10_ cfu/thigh compared with bacterial load before initiation of treatment (Δlogcfu) in infected mice. The dotted line at 0 Δ^10^logcfu represents bacterial stasis, while the broken line at 1 Δ^10^logcfu indicates 1-logkill.

The dose–response relationship of cefepime alone followed a sigmoid pattern (R^2^ ≥ 0.73) (Figure 2). Stasis was reached for all strains, but 1 log_10_ kill effect was found only for the VIM-producing *P. aeruginosa* 132 isolate within the doses tested. The corresponding doses and PK/PD indices for a stasis and 1 log_10_ kill effect for each strain are shown in Table 2.

**Table 2.** Estimated doses and PK/PD targets of Enterobacterales and *P. aeruginosa* strains used for thigh infections in neutropenic mice treated with cefepime monotherapy (numbers in parentheses are extrapolated values as stasis and 1 log_10_ kill effect was not found within the tested doses)

Strain no.	Cefepime MIC, mg/L	Stasis^a^			1 log_10_ kill^a^		
		Total daily dose, mg/kg	q2h dose, mg/kg	%ƒT > MIC	Total daily dose, mg/kg	q2h dose, mg/kg	%ƒT > MIC
*E. coli* 52	32	(1857.2)	(154.8)	(36.5)	(5237.2)	(436.4)	(53.9)
*K. pneumoniae* 59	256	1413.4	117.8	0.0	(4371.3)	(364.0)	15.9
*P. aeruginosa* 106	32	(1901.6)	(158.5)	(36.9)	(3484.4)	(290.4)	(47.4)
*P. aeruginosa* 132	16	180.8	15.5	8.9	718.3	59.9	32. 4

^a^Stasis and 1 log kill were not observed within the tested doses from some strains. Therefore, doses were extrapolated beyond the maximum dose used in the present study using the E_max_ model based on the dose–response data obtained with lower doses.

### Pharmacodynamics of taniborbactam in combination with cefepime

**Figure 3.**
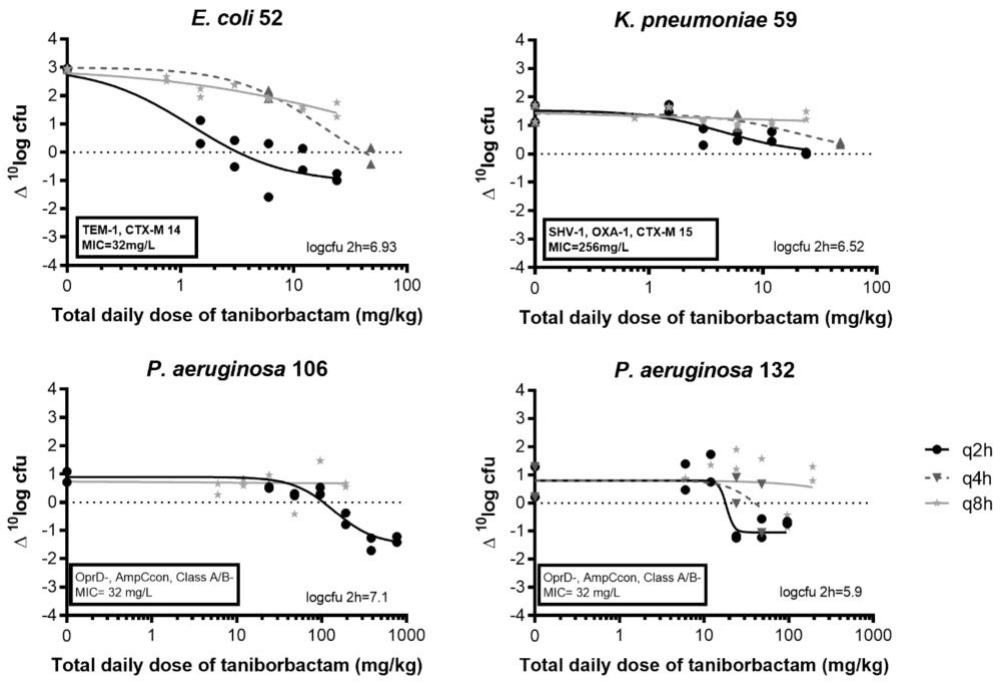
Dose fractionation effects of taniborbactam in combination with q2h cefepime dosing regimen for *E. coli* 52, *K. pneumoniae* 59, and *P. aeruginosa* 106 and 132.

**Figure 4.**
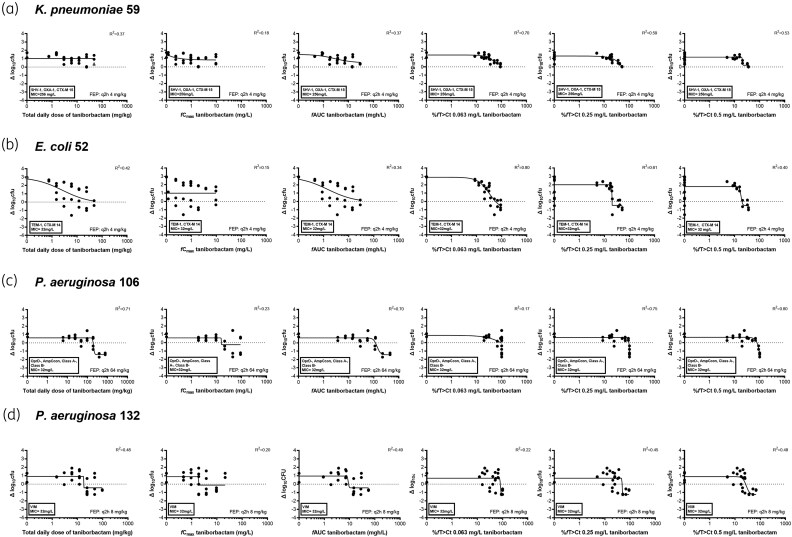
Exposure–response relationships of taniborbactam in dose fractionation experiments for two Enterobacterales (a, b) and two *P. aeruginosa* (c, d) with the cefepime dosing regimens as indicated at the bottom right of the graphic. *f*AUC, area under the concentration–time curve for free drug; *f*C_max_, maximum concentration in plasma for free drug; FEP, cefepime.

The exposure–response curves of q2h, q4h and q8h for all isolates are shown in Figure 3. The q2h regimen was more effective than q8h as the ED_50_ values were statistically significantly lower in the following order: q2h < q4h < q8h for *E. coli* 52 (*P* = 0.046), *K. pneumoniae* 59 (*P* < 0.0001), *P. aeruginosa* 106 (*P* = 0.0008) and *P. aeruginosa* 132 (*P* = 0.0008). When different PK parameters (dose, *f*C_max_, *f*AUC and %*f*T > Ct) were analysed with the E_max_ model (Figure 4), best fits were found with the %*f*T > Ct. Among the different Cts analysed, the highest R^2^ was found for %*f*T > Ct for a Ct of 0.03–0.06 mg/L for *E. coli* 52 (R^2^ = 0.79–0.80), 0.06–0.125 mg/L for *K. pneumoniae* 59 (R^2^ = 0.70–0.71), 0.5–4 mg/L for *P. aeruginosa* 106 isolate (R^2^ 0.79–0.80) and 0.25–1 mg/L for *P. aeruginosa* 132 isolate (R^2^ = 0.48–0.50) (Table 3). For. *P. aeruginosa* isolates, compared with %*f*C > Ct, relative good fits were found with *f*AUC for *P. aeruginosa* 106 isolate (R^2^ = 0.70) and for *P. aeruginosa* 132 isolate (R^2^ = 0.49).

**Table 3.** R^2^ of taniborbactam exposure–response relationship for each strain for total daily dose, *f*C_max_, *f*AUC and %*ƒ*T > Ct at different Cts (0.03–4 mg/L) when combined with cefepime q2h dosing regimens. In bold are shown the highest R^2^.

	Cefepime MIC, mg/L	Cefepime q2h dose, mg/kg	R^2^										
Strain no.			TDD	*f*C_max_	*f*AUC	%ƒT > Ct at the following taniborbactam Ct:							
						0.03	0.06	0.125	0.25	0.5	1	2	4
*E. coli* 52	32	4	0.42	0.15	0.34	0.79	**0**.**80**	0.66	0.61	0.40	0.28	0.27	0.08
*K. pneumoniae* 59	256	4	0.37	0.18	0.37	0.61	0.70	**0**.**71**	0.59	0.53	0.44	0.12	0.12
*P. aeruginosa* 106	32	64	0.71	0.24	0.70	0.16	0.16	0.42	0.75	0.80	**0**.**80**	0.79	0.79
*P. aeruginosa* 132	16	8	0.48	20	0.49	0.22	0.45	0.45	0.48	0.48	**0**.**50**	0.24	0.05

Ct, concentration threshold; *f*C_max_, maximum free drug concentration; *f*AUC, area under the free concentration–time curve; %*f*T > Ct, percentage of time above concentration threshold; TDD, total daily dose.

The magnitudes of %*f*T > Ct of taniborbactam q2h dosing regimens in combination with cefepime q2h at different Cts for stasis and 1 log_10_ kill for all strains are shown in Table 4. For Enterobacterales, the %*f*T > Ct for Cts 0.06 mg/L and 0.125 mg/L associated with stasis was 36.6% and 19.7% for ESBL-producing *E. coli* 52, and 86.2% and 69.3% for the ESBL-producing *K. pneumoniae* 59, respectively. Between the two strains, only *E. coli* 52 reached 1 log_10_ kill effect at %*f*T > Ct for Cts 0.06 and 0.125 mg/L of 99.7% and 82.8%, respectively. For the *P. aeruginosa* strains, the %*f*T > Ct for Ct 1 mg/L associated with stasis was 54.6% for *P. aeruginosa* 106 and 11.6% for *P. aeruginosa* 132. The 1 log_10_ kill effect was found for both *P. aeruginosa* 106 and 132 at 79.6% and 20.6% %*f*T > Ct 1 mg/L, respectively. The %*f*T > Ct associated with stasis for Cts where reversal of cefepime resistance was found *in vitro* (i.e. at ≤0.03 mg/L for *E. coli*, 0.25 mg/L for *K. pneumoniae*, 0.125 mg/L for the VIM-producing *P. aeruginosa* and 2 mg/L for AmpC constitutively-producing *P. aeruginosa*) were more uniform. The %*f*T > Ct for stasis and 1 log kill was ∼50% and 100% for Enterobacterales and ∼40% and ∼60% for *P. aeruginosa* isolates, respectively.

**Table 4.** Magnitude of %*f*T > Ct of taniborbactam for stasis and a 1 log_10_ kill for different Cts when combined with cefepime

Isolate no.	Cefepime MIC, mg/L	Cefepime potMIC,^a^ mg/L	Cefepime q2h dose, mg/kg	Cefepime %*f*T > MIC	Cefepime %*f*T > potMIC^a^	Taniborbactam total daily dose, mg/kg	Taniborbactam %ƒT > Ct, mg/L								
							0.015	0.03	0.06	0.125	0.25	0.5	1	2	4
**Stasis**															
*E. coli* 52	32	0.06	4	0.00	80.70	3.20	70.24	53.44	36.55	19.66	2.74	0	0	0	0
*K. pneumoniae* 59	256	0.5	4	0.00	69.30	24.00	100	100	86.2	69.3	52.4	35.5	18.6	0	0
*P. aeruginosa* 106	32	4	64	22.10	57.70	105.00	100	100	100	100	88.39	71.5	54.61	37.74	20.82
*P. aeruginosa* 132	16	1	8	0.00	46.10	18.00	100	96.10	79.2	62.31	45.1	28.53	11.63	0	0
**1 log kill**															
*E. coli* 52	32	0.06	4	0.00	80.70	41.8	100	100	99.7	82.82	65.92	49.3	32.14	15.24	0
*K. pneumoniae* 59	256	0.5	4	0.00	69.30	(680)^b^	100	100	100	100	100	100	99.01	83.25	66.35
*P. aeruginosa* 106	32	4	64	22.10	57.70	293	100	100	100	100	100	96.5	79.61	62.71	45.8
*P. aeruginosa* 132	16	1	8	0.00	46.10	26	100	100	88.19	71.30	54.41	37.51	20.62	3.72	0

Ct, threshold concentration; %*f*T > Ct, percentage of time that free concentration remained above threshold concentration.

^a^Potentiated MIC (potMIC) is the MIC of cefepime in the presence of 4 mg/L taniborbactam.

^b^Number in parentheses corresponds to extrapolated dose as the corresponding effect was not reached within the tested doses.

## Discussion

*In vitro*, reversal of phenotypic resistance to cefepime was found at taniborbactam ≤0.03 and 0.25 mg/L for the ESBL-producing *E. coli* and *K. pneumoniae* strains, and 0.125 and 2 mg/L for the VIM- and the AmpC constitutively -producing *P. aeruginosa* isolates, respectively. *In vivo*, cefepime alone marginally reached stasis, and 1 log kill was found only for the VIM-producing *P. aeruginosa* isolates. When marginally ineffective doses of cefepime were combined with taniborbactam, cefepime regained its static effect against all isolates at taniborbactam TDDs of 3.2–105 mg/kg and its 1 log_10_ kill effect against all strains except *K. pneumoniae* at TDDs of 26–293 mg/kg. Taniborbactam enhanced cefepime’s activity in a time-dependent manner as q2h regimens were >10-fold more effective than q8h regimens. For *P. aeruginosa* where higher cefepime exposures were used (48 and 768 mg.h/L), the fit with *f*AUC was equally well as %*f*T > Ct, indicating a concentration-dependent action. Stasis was associated with a %*f*T > Ct of ∼50% for ESBL-producing Enterobacterales and 40% for *P. aeruginosa* isolates at Cts where *in vitro* reversal of cefepime resistance was found, i.e. ≤ 0.03 mg/L for *E. coli*, 0.25 mg/L for *K. pneumoniae*, and 0.125 mg/L for the VIM-producing and 2 mg/L for the AmpC constitutively-producing *P. aeruginosa* isolates. At the latter Cts and for 1 log_10_ kill effect a %*f*T > Ct of 100% for Enterobacterales and ∼60% for *P. aruginosa* was required.

Taniborbactam exhibited an MIC- and exposure-dependent time-dependent activity when combined with cefepime, with q2h dosing regimens being more effective than q8h dosing regimens. Although for Enterobacterales the E_max_ model fit was significantly better with %*f*T > Ct than *f*AUC (R^2^ = 0.70–0.80 versus 0.34–0.37, respectively) indicating strong time-dependent activity; for *P. aeruginosa* the %*f*T > Ct gave slightly better fit than *f*AUC (R^2^ = 0.48–0.5 versus 0.49 for one isolate and 0.75–0.8 versus 0.70 for the second isolate, respectively) indicating some concentration-dependent action. Of note, the potentiated cefepime MIC for the two *P. aeruginosa* isolates was 1 and 4 mg/L whereas for the Enterobacterales it was 0.06 and 0.5 mg/L; cefepime exposures tested in combination were higher for *P. aeruginosa* than Enterobacterales (*f*AUC_0-24_ 48–338 versus 24 mg.h/L, respectively). An AUC-dependent activity was concluded in previous animal studies with a thigh infection model against two KPC strains (one *K. pneumoniae* and one *P. aeruginosa* with unpotentiated and potentiated cefepime MIC >512 and >512 mg/L, and 2 and 4 mg/L, respectively) using a human simulated regimen of cefepime 2 g q8h as 2 h infusion (*f*AUC_0-24_ ∼787.8 mg.h/L), higher than the cefepime exposures combined with taniborbactam in the present study (*f*AUC_0-24_ 24–384 mg.h/L).^26^ It seems that taniborbactam acts in a time-dependent manner at low cefepime exposures against isolates with a potentiated cefepime MIC <1 mg/L, and in a concentration-dependent manner at higher cefepime exposures against isolates with a potentiated cefepime MIC ≥1 mg/L. *In vitro* PK/PD studies where human simulated regimens were used also found that both AUC and *f*T > Ct were predictive indices for taniborbactam efficacy.^27,28^ Interestingly, most of the isolates had potentiated cefepime MICs >1 mg/L. At low cefepime exposures more frequent administration of taniborbactam may be needed to enhance cefepime’s activity against resistant Gram-negative bacteria whereas at high cefepime exposures, which possess some antibacterial activity, high exposures of taniborbactam are required to protect cefepime from enzymatic degradation by β-lactamases. Low cefepime exposures may be observed in patients with augmented clearance, those who are obese, who are undergoing renal replacement therapy, and in certain tissues like CSF.^29^ Alternatively, as cefepime/taniborbactam’s activity is affected by different resistance mechanisms such as β-lactamases, PBP3 changes and efflux pumps,^30^ inhibition of these mechanisms may be time- and concentration-dependent at different exposures depending on the contribution of each mechanism to resistance.

No kill was observed for the *K. pneumoniae* isolate within the tested doses. The *K. pneumoniae* isolate had the highest MIC of cefepime, possibly due to underlying resistance mechanisms (OXA-1, SHV-1, CTX-M-15). Another interesting observation is the activity of taniborbactam against MBL producers and particularly *P. aeruginosa* in this study, where exposures for stasis and 1 log_10_ kill were similar to those against ESBL-producing Enterobacterales. Taniborbactam has been shown to restore cefepime susceptibility of ESBL-producing Enterobacterales^23^ and MBL-producing *P. aeruginosa* isolates,^21^ whereas against AmpC constitutively-producing *P. aeruginosa* the enhancement was weaker, reducing cefepime MICs by a 2-fold dilution compared with a ≥2-fold MIC reduction for MBL- and ESBL-producing isolates.^23^ PK studies in healthy adults indicate that these PD targets are achievable in humans^31,32^ with a taniborbactam dose of 500 mg infused over 2 h three times daily reaching a total C_max_ of 22 mg/L and half-life of 10 h, resulting in 100% *f*T > Ct 8 mg/L attaining even the highest 1 log_10_ kill PK/PD targets for *P. aeruginosa*. Compared with other combinations of a cephalosporin with β-lactam inhibitors, ceftazidime/avibactam and ceftolozane/tazobactam, the Cts were 1 mg/L^8^ for avibactam and 0.5 mg/L^24^ for tazobactam, indicating that a lower Ct of taniborbactam is required to produce the same effect.

In conclusion, taniborbactam enhanced cefepime’s *in vivo* efficacy mainly in a time-dependent manner although at high cefepime exposures a concentration-dependent action was observed. A taniborbactam %*f*T > Ct of ∼40%–50% for ESBL- and MBL-producing isolates is required for stasis for Ct 0.03–0.25 mg/L for Enterobacterales and VIM-producing *P. aeruginosa*, and 2 mg/L for AmpC constitutively-producing *P. aeruginosa* isolates. For 1 log_10_ kill effect 60%–100% *f*T > Ct was required with the same Ct values. These targets can be used in order to further determine optimal human doses and clinical breakpoints, particularly for clinical scenarios where low cefepime exposures are expected.

## Supplementary Material

dkaf431_Supplementary_Data

## Supplementary data

Figures S1 and S2 and Table S1 are available as Supplementary data at *JAC* Online.
