# Supplementary material for: Pharmacodynamic studies of taniborbactam (VNRX-5133) combined with cefepime against β-lactamase–producing Gram-negative bacteria in a neutropenic murine thigh infection model
Source: J Antimicrob Chemother. 2025 Dec 5;81(1):dkaf431. doi: 10.1093/jac/dkaf431 (PMC12802961; doi:10.1093/jac/dkaf431)
Supplement: dkaf431_Supplementary_Data [file dkaf431_supplementary_data.docx]

**Supplementary material**

**Population pharmacokinetic model of taniborbactam**

For taniborbactam, a population pharmacokinetic model was developed using nonlinear mixed-effects modelling (NONMEM). The analysis was performed on the natural logarithm of the concentrations using the FOCE method with INTERACTION. All mice were assumed to weigh 1kg, resulting in PK parameters corresponding to a per kg base. All fitting procedures were performed with the use of NONMEM v7.2 (ICON Development Solutions, Ellicott City, MD, USA) and Intel Visual Fortran Compiler XE 2013 update 5 (Santa Clara, CA, USA). Tools used to evaluate and visualize the model were RStudio (v0.98.1049), R (v3.1.2), XPose (v4.5.3) and PsN (v4.6.0), all with the graphical interface Pirana (v2.9.0).

In the first step a structural model was developed to describe the pharmacokinetics of taniborbactam-5133. To determine the structural pharmacokinetic model 1-, 2- and 3-compartment models were tested. Pharmacokinetic parameters were estimated in terms of central and peripheral volume of distribution (Vc and Vp), clearance (CL), and intercompartmental clearance (Q). Addition of interindividual variability (IPV), described using an exponential eta model, was evaluated for each pharmacokinetic parameter. The covariance between values for IPV was estimated using a variance–3covariance matrix. Residual variability between observed and predicted plasma concentrations was described using an additional error model for logarithmically transformed data. The minimum value of objective function (OFV) was used as a criterion for model selection. If the difference between two nested models was larger than the critical value from a Χ2 distribution with degrees of freedom equal to the difference in the number of estimated parameters, the models were significantly different from each other. A decrease in OFV>10.83 showed a significant improvement of a nested model with one degree of freedom of p<0.001. Model adequacy was further evaluated by using various residual plots (‘goodness-of-fit’ plots) and values of random effects in variances. In the second step relationships between pharmacokinetic parameters and potential covariates were investigated. Covariates assessed were place of infection and dose of taniborbactam and cefepime. Dose covariates were modelled by using an exponential model, place of infection was modelled proportionally. Criteria for the inclusion of covariates in the model were the OFV, results of graphical evaluation of the parameter-covariate relationship and the decrease in parameter variability.

Three procedures were used to validate the final model. As an internal validation method, a bootstrap resampling method was used. Bootstrap datasets (n=1000) were generated by sampling randomly from the original dataset with replacement. Parameters were estimated for each of the replicate datasets using the developed model. The validity of the model was evaluated by comparing the median values and 95% percentiles of the bootstrap replicates with the estimates of the original dataset. The predictive performance of the model was evaluated using a visual predictive check (VPC). The original data obtained in the mice was compared to 1000 datasets simulated using the final model. The results of these analyses were corrected for the different doses using the option ‘-predcorr’ in PsN.

Serum pharmacokinetics followed a two-compartment model **(Table S1, Figure S1 and S2)**. Addition of the second compartment resulted in a graphical improvement of the model and a decrease in OFV of 146 points. Addition of a third compartment did not further improve the model evaluated by OFV, goodness-of-fit plots and NPDE. Due to the limited information available in the absorption phase, Ka was fixed at a value of 20. Other values were tested but did not result any improvement. Addition of IPV on CL and Q resulted in significant improvement of the model. Evaluation of the covariates on CL and Q did not result in a significant correlation. The parameter estimates are presented in **Table S1**. **Figure S1** shows the goodness-of-fit plots of the final model. No structural deviation was seen. Finally, the model was validated using two techniques. Firstly, bootstrap analysis of 772 successful runs was performed (**Table 2**). This analysis showed that the parameters were well estimated. The 95% CI of Vp and Q show a wider range, which indicates the estimation of these parameters is less accurate based on the current data. Last, the dose corrected VPC shows (**Figure S2**) that the observed data are estimated well by the simulated data. Pharmacokinetics were similar for the two infection models.

**Table S1:** Parameter estimates of the taniborbactam-model. Estimations are presented as value (RSE) per kg. Bootstrap represents median and 95-percentile range of 772 successful bootstrap runs.

| **Parameter** | **Estimation** | **Shrinkage (%)** | **Bootstrap** |
| --- | --- | --- | --- |
| **Absorption rate (h-1)** | 20 (fixed) |  | 20 |
| **Central volume of distribution (L/Kg)** | 0.628 (4%) |  | 0.63 (0.58-0.68) |
| **Clearance (L/h/Kg)** | 1.49 (3%) |  | 1.49 (1.23-1.57) |
| **IPV (%)** | 14.2 (14%) | 36 | 14 (8-19) |
| **Peripheral volume of distribution (L/Kg)** | 0.926 (1%) |  | 0.94 (0.46-13.0) |
| **Intercompartmental clearance (L/h)** | 0.109 (10%) |  | 0.11 (0.08-0.35) |
| **IPV (%)** | 60.7 (13%) | 46 | 59 (40-77) |
| **Residual error** | 0.155 (15%) | 46 | 0.15 (0.08-0.21) |


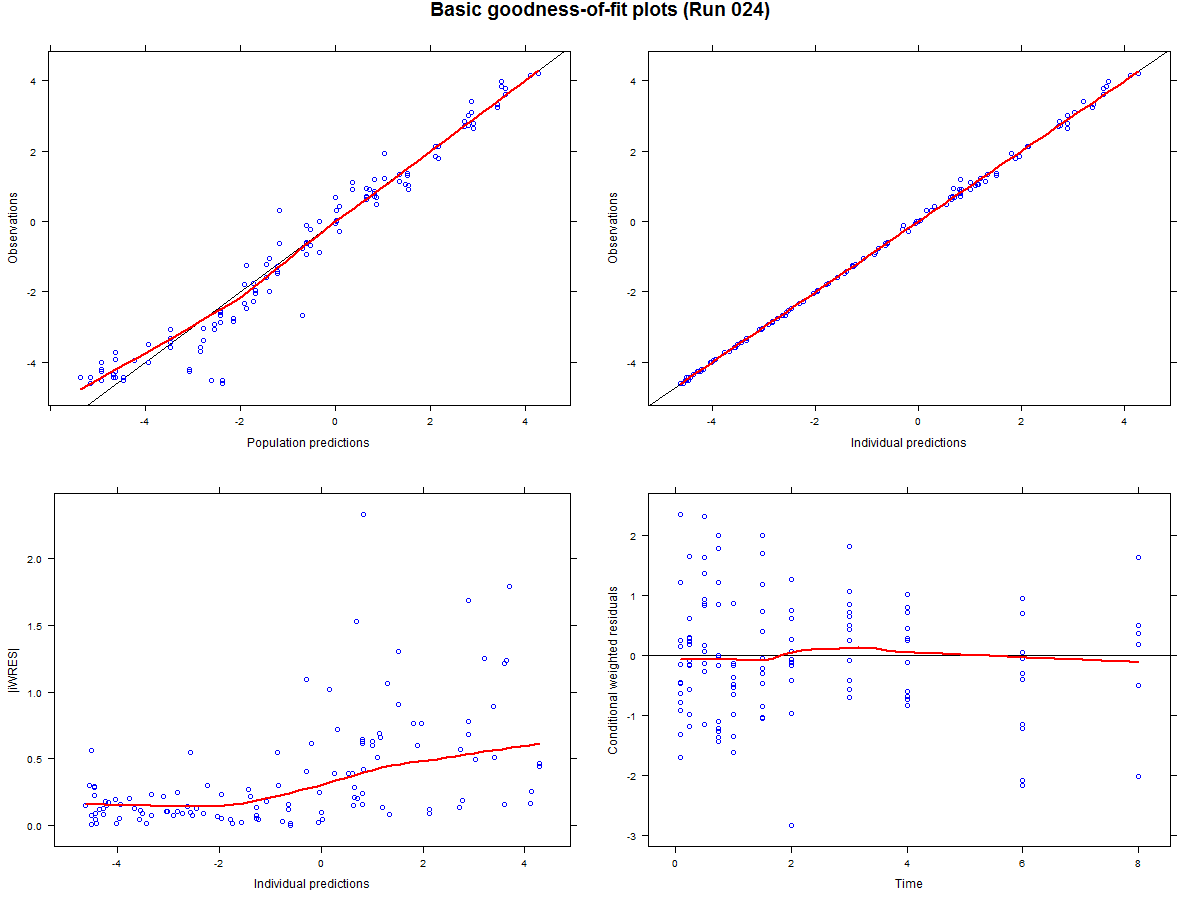


**Supplementary Figure 1:** Goodness-of-fit plots of the final taniborbactam model


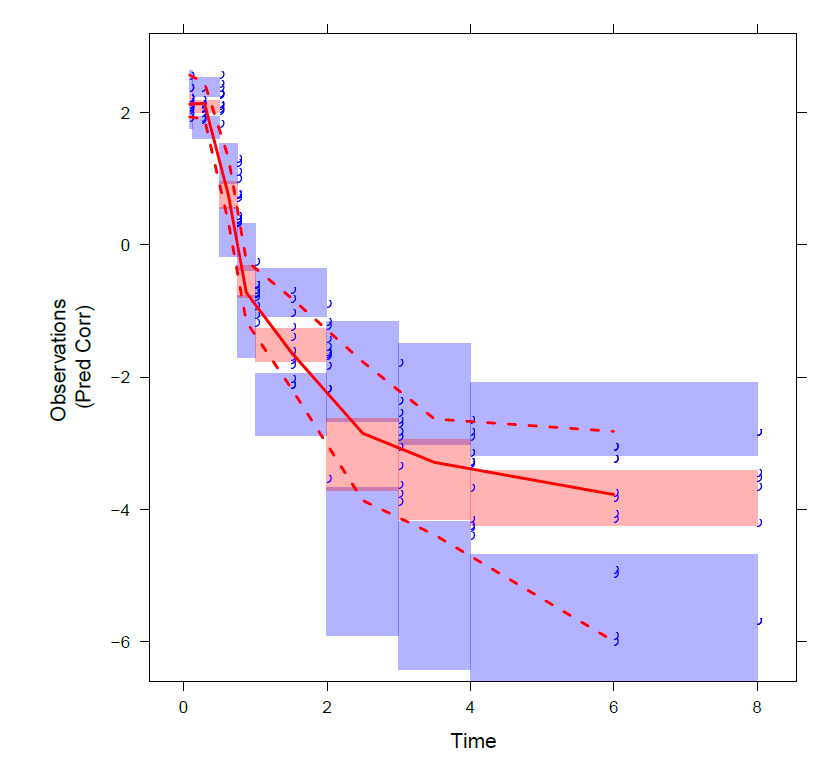


**Supplementary Figure 2:** Dose corrected visual predictive check for the taniborbactam model. The observations are presented as the natural logarithm of the concentration versus the time in hours. The red lines represent the mean and 95%CI of the observations, the colored areas represent the 95%CI of the mean and 95%CI of 1000 simulated datasets**.**
